# Standalone oblique lateral interbody fusion vs. combined with percutaneous pedicle screw in spondylolisthesis

**DOI:** 10.1186/s12891-020-03192-7

**Published:** 2020-03-23

**Authors:** Wei He, Da He, Yuqing Sun, Yonggang Xing, Jiankun Wen, Weiheng Wang, Yanhai Xi, Mingming Liu, Wei Tian, Xiaojian Ye

**Affiliations:** 1 Department of Spine Surgery, Beijing Jishuitan Hospital, No.31 Xinjiekou East Street, Xicheng District, Beijing, 100035 China; 2 Department of Spine Surgery, Shanghai Changzheng Hospital, No.415, Fengyang road, Huangpu District, Shanghai, 200003 China

**Keywords:** Spondylolisthesis, Oblique lumbar interbody fusion, Percutaneous pedicle screw fixation, Radiological outcomes

## Abstract

**Background:**

To compare standalone oblique lateral interbody fusion (OLIF) vs. OLIF combined with posterior bilateral percutaneous pedicle screw fixation (OLIF combined) for the treatment of lumbar spondylolisthesis.

**Methods:**

This was a retrospective study of patients who underwent standalone OLIF or combined OLIF between 07/2014 and 08/2017 at two hospitals in China. Direct decompressions were not performed. Visual analog scale (VAS), Oswestry Disability Index (ODI), satisfaction rate, anterior/posterior disc heights (DH), foraminal height (FH), foraminal width (FW), cage subsidence, cage retropulsion, fusion rate, and complications were analyzed. All imaging examinations were read independently by two physicians and the mean measurements were used for analysis.

**Results:**

A total of 73 patients were included: 32 with standalone OLIF and 41 with combined OLIF. The total complication rate was 25.0% with standalone OLIF and 26.8% with combined OLIF. There were no differences in VAS and ODI scores by 2 years of follow-up, but the scores were better with standalone OLIF at 1 week and 3 months (*P* < 0.05). PDH and FW was smaller in the combined OLIF group compared with the standalone OLIF group before and after surgery (all *P* < 0.05). There were significant differences in FH before surgery and at 1 week and 3 months between the two groups (all *P* < 0.05), but the difference disappeared by 2 years (*P* = 0.111). Cage subsidence occurred in 7.3% (3/41) and 7.3% (3/41) of the patients at 3 and 24 months, respectively, in the combined OLIF group, compared with 6.3% (2/32) and 15.6% (5/32), respectively, in the standalone OLIF group at the same time points (*P* = 0.287). There was no cage retropulsion in both groups at 2 years. The fusion rate was 85.4%(35/41) in the combined OLIF group and 84.4% (27/32) in the standalone OLIF group at 3 months(*P* = 0.669). At 24 months, the fusion rate was 100.0% in the combined OLIF group and 93.8% (30/32) in the standalone OLIF group (*P* = 0.066).

**Conclusion:**

Standalone OLIF may achieve equivalent clinical and radiological outcomes than OLIF combined with fixation for spondylolisthesis. The rate of complications was similar between the two groups. Patients who are osteoporotic might be better undergoing combined rather than standalone OLIF. The possibilty of proof lies within a future prospective study, preferably an RCT.

## Background

Degenerative lumbar spine disease (DSD) is an important cause of disability, affecting 3.6% of the world population. The prevalence of DSD is four times higher in low-and middle-income countries compared with high-income countries [1].

Fusionis the corners to neither treatment of an unstable degenerative lumbar spinal disease, but various techniques are available [2, 3]. Oblique lateral interbody fusion (OLIF) is are gently introduced surgical technique [4] that evolved from lateral lumbar interbody fusion (LLIF) and anterior lumbar interbody fusion (ALIF) [5]. To avoid femoral nerve plexus injury during the transpsoas approach in LLIF, OLIF use a natural space between the left lateral border of the abdominal aorta (AA) and the anterior medial border of the left psoas muscle (PM), all owing access to the lesion discs from L2/3 to L4/5 without splitting the left PM [6]. OLIF has advantages over LLIF in that the larger lateral cage can achieve greater fusion rate and angular correction. This surgical procedure alleviate spost operative back pain, shortens the time of operation and hospitalization, and also reduces bleeding compared with open surgery [5, 6].

It remains controversial as whether internal fixation is required for OLIF [7]. The standalone OLIF approach is associated with small trauma and short operation time and hospital stay [8–11]. Zeng et al. [7] suggested that the rate of complications was lower with the use of combined screw fixation. Posterior pedicle screw fixation can be indicated inpatients with endplate damage [7]. Ohtori et al. [12] used posterior screws in all their patients and reported good outcomes. Studies comparing standalone OLIF and OLIF combined with pedicle screws are lacking inpatients with spondylolisthesis.

Considering the emerging interest for OLIF, the present study aimed to analyze the clinical experience with standalone OLIF and OLIF combined with pedicle screws, and to compare their clinical and radiological outcomes in the management of primary spondylolisthesis.

## Materials and method

### Study design and patients

This was a retrospective study of patients who underwent standalone OLIF or OLIF combined with posterior bilateral percutaneous pedicle screw fixation (combined OLIF group) between July 2014 and August 2017 at the Beijing Jishuitan Hospital (combined OLIF group) and the Shanghai Changzheng Hospital (standalone OLIF group). Only one type of procedure was performed at each hospital. The study was approved by the ethical committees of the Beijing Jishuitan Hospital (approval number: 201811–03) and the Shanghai Changzheng Hospital (approval number: 201812–01). The need for individual consent was waived by both committees because of the retrospective nature of the study.

Lumbar spondylolisthesis was diagnosed according to plain X-ray examination [13]. The inclusion criteria were: 1) patients who underwent surgery at the L4–5 level; 2) diagnosis of spondylolisthesis with symptoms; 3) grade I spondylolisthesis (< 25%) based on Meyerding classification; 4) failure to > 6 months of conservative treatment; 5) no history of lumbar surgery at L4–5; and 6) > 24 months of follow-up. The exclusion criteria were: 1) lumbar spondylolysis; 2) lumbar canal stenosis; 3) lumbar disc herniation; or 4) incomplete medical records.

### Surgical techniques

Surgeries were performed by chief physicians with > 20 years of surgical experience in both hospitals. In the standalone OLIF group, surgery was performed based on standard procedure [5, 6]. Presence of scoliosis did not affect the side of the surgical approach. A4-cm skin incision was made 6-10 cm anterior to the mid-portion of the marked disc. The surgeons approached the retroperitoneal space by blunt dissection and mobilization of the peritoneum anteriorly to expose the anatomical oblique lateral corridor, followed by intervertebral cage insertion (Clydesdale spinal system, Medtronic, Memphis, TN, USA; 12 mm in height× 50 mm in length× 18 mm in width, 6°lordotic, 3.27 cc graft volume) filled with demineralized bone matrix DBM (Wright Medical Technology Inc., Arlington, TN, USA). Direct decompressions were not performed.

In the combined OLIF group, OLIF surgery was performed based on the standard procedure [5, 6], as above. After fusion, the patients were placed in the prone position to undergo posterior bilateral percutaneous pedicle screw fixation (CD Horizon Solera Voyager Spinal System, Medtronic, Memphis, TN, USA). None of the patients in either group underwent additional laminectomy.

Non-steroidal drugs were used for postoperative analgesia with muscle relaxants. The back muscle function was exercised by swimming and agymnastic named “sky diver to superman to swimmer”, as recommended by the North American Spine Society (NASS).

### Data collection

The demographic data included sex, age, bone mineral density (BMD), and fixation level. BMD was measured by dual-energy X-ray absorptiometry (DEXA). The T-score is the BMD when compared to the young normal reference mean. T < -2.5 was defined as osteoporosis. The patients’ clinical results were routinely assessed based on visual analog scores (VAS) for leg pain and the Oswestry disability index (ODI,version2.0) at 1 week, 3 months, and 24 months. The patients were surveyed for satisfaction: satisfied, unsatisfied, and feeling worse than before surgery. All patients underwent routine preoperative and postoperative standing anteroposterior (AP) /lateral plain X-ray, flexion-extension plain X-ray, and computed tomography (CT). Assessments were done for cage subsidence and cage retropulsion. As the true footprint of the cage can be determined on CT images, CT images were used to analyze the cage position. Cage subsidence was evaluated using postoperative and serial follow-up sagittal CT images, and was defined as a cage sinking into an adjacent vertebral body by > 2 mm, based on comparisons with previous CT images [14]. Cage migration was defined as posterior movement of the cage by ≥3 mm compared with the immediate postoperative estate. These data were collected before surgery, and at 1 week, 3 months, and 2 years postoperatively. In addition, complications were recorded.

### Lumbar radiological measurements

**Fig. 1:**
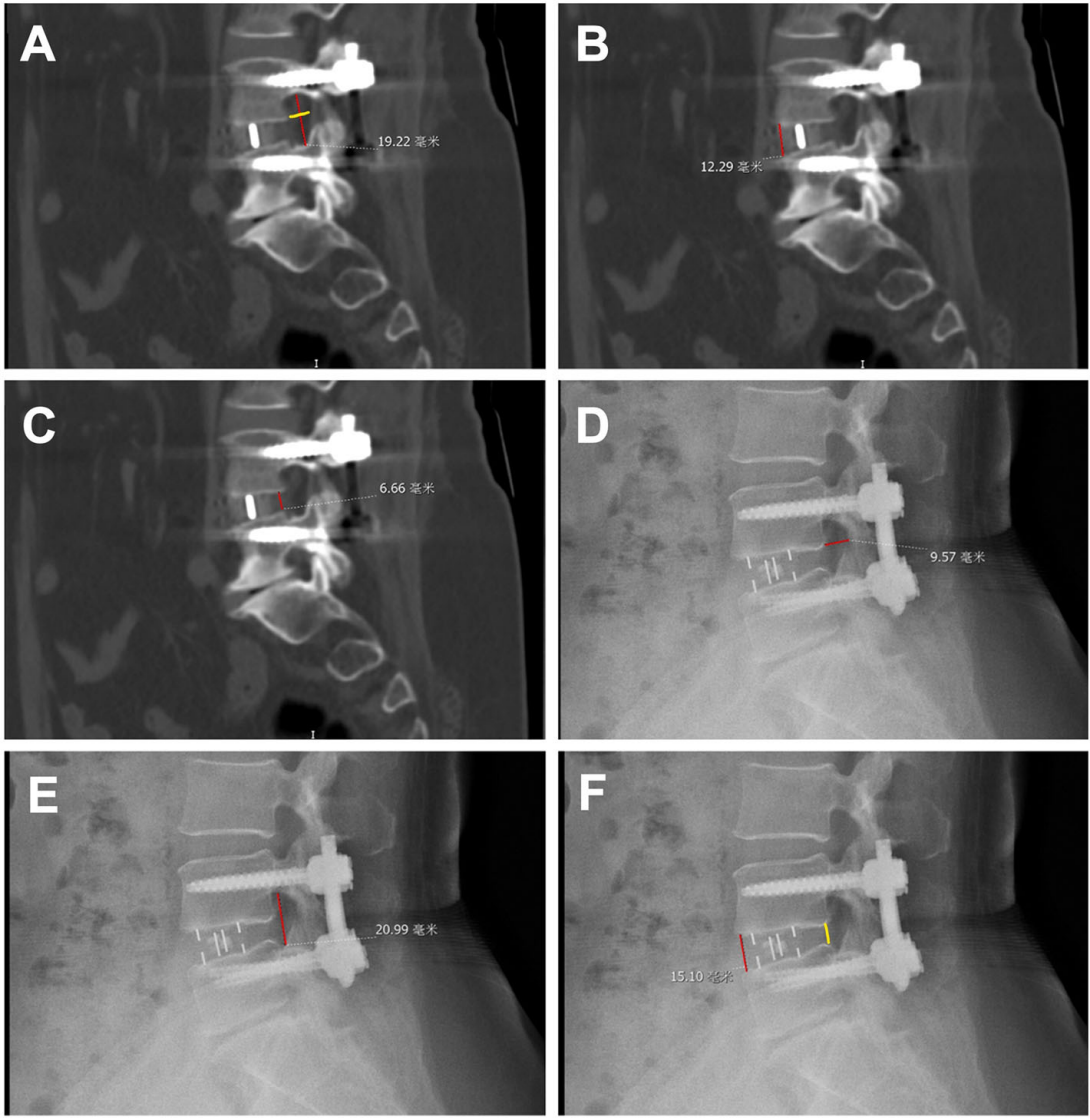
Radiological measurements (CT and X ray) used in this study. **a** Sagittal computed tomography (CT) image of lumbar vertebrae. The red line shows the foraminal height (FH). The yellow line shows the foraminal width (FW). **b** Sagittal CT image of lumbar vertebrae. The red line shows the anterior disc height (ADH), (**c**) Sagittal CT image of lumbar vertebrae. The red line shows the posterior disc height (PDH), (**d**) Lateral X-ray image of lumbar vertebrae. The red line shows the FW. **e** Lateral X-ray image of lumbar vertebrae. The red line shows the FH. **f** Lateral X-ray image of lumbar vertebrae. The red line shows the ADH. The yellow line shows the PDH

Figure 1 presents the radiological measurements. Radiological outcomes were measured on lateral X-rays and sagittal CT images, including anterior/posterior disc heights (ADH/PDH), foraminal height (FH), foraminal width (FW), fusion rate, and cage subsidence. All imaging examinations were read independently by two physicians (at least 12 years of experience) who were blind to grouping and the mean measurements were used for analysis. The calculated intra-class correlation coefficients were all > 0.85 for all variables. CT slice thickness was 2 mm, as per routine practice. ADH/PDH was defined as the distance at the anterior/posterior position from upper to lower endplate of the L4–5 level. FH was defined as the distance from the lower position of the pedicle of L4 to the upper position of the pedicle of L5. FW was defined as the distance from the lower posterior horn of the vertebral body of L4 to the vertex of the superior joint of L5. The fusion rate grading criteria were based on the Bridwell interbody fusion grading system [15]. Grades I and II were considered as successful.

### Statistic analysis

Statistical analysis was performed using SPSS 18.0 for Windows (IBM, Armonk, NY, USA). Continuous data with normal distribution are presented as means ± standard deviation, and were analyzed using the Student t test. Continuous data with non-normal distribution are presented as medians (range), and were analyzed using the Wilcoxon test. Categorical variables are presented as frequencies, and were analyzed using the Pearson chi-square test or the Fisher exact test, as appropriate. Two-tailed *P* values< 0.05 were considered statistically significant.

## Results

### Characteristics of the patients and clinical outcomes

The characteristics of the patients are presented in Table 1. A total of 73 patients were included: 32 with standalone OLIF and 41 with combined OLIF.

**Table 1:** Baseline and clinical data of the patients according to the surgical procedure they underwent

	OLIF+fixation	Standalone OLIF	P
N	41	32	
Sex			0.679
Male	11 (26.8%)	10 (31.3%)	
Female	30 (73.2%)	22 (68.7%)	
Age (years)	61.0 ± 9.3	59.8 ± 13.7	0.669
Osteoporosis	15 (36.6%)	8 (25.0%)	0.290

**Fig. 2:**
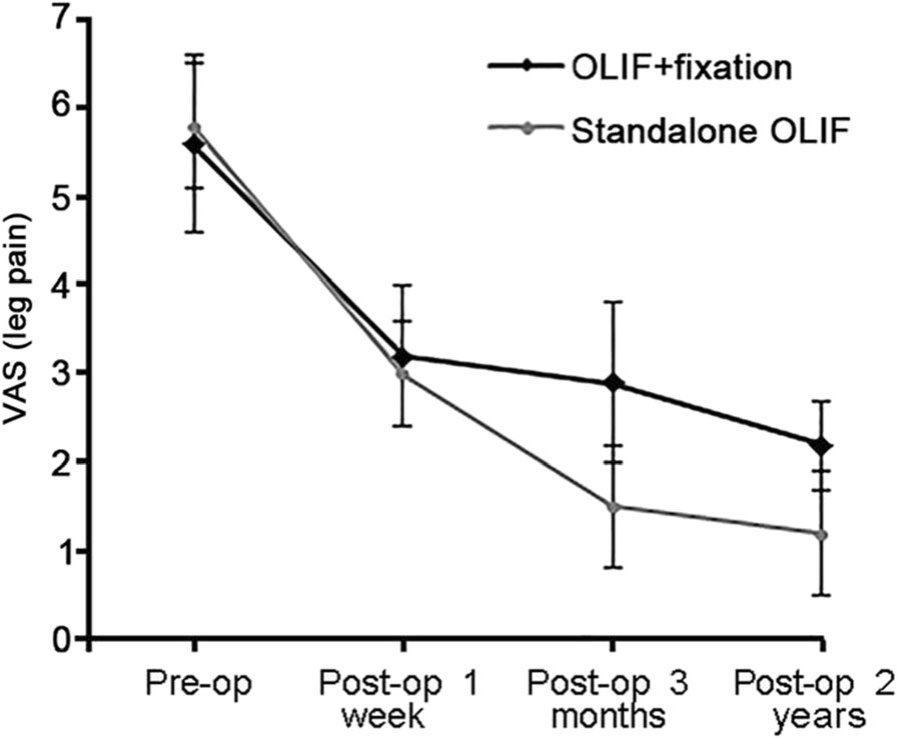
Visual analogue scale of leg pain. Comparison between standalone oblique lateral interbody fusion (OLIF) and OLIF combined with pedicle screw fixation, from base line to 24 months after surgery

**Fig. 3:**
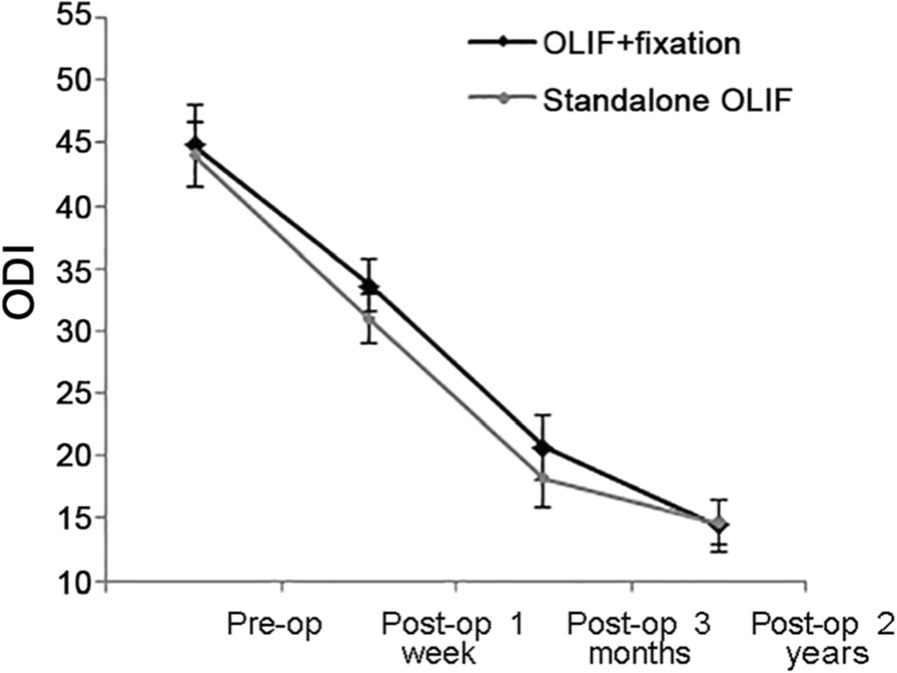
Oswestry disability index. Comparison between standalone oblique lateral interbody fusion (OLIF) and OLIF combined with pedicle screw fixation, from base line to 24 months after surgery

Satisfaction with surgery was 87.5% with standalone OLIF and 92.7% with combined OLIF (*P* = 0.692). Patients in the standalone OLIF group had better ODI scores than patients in the combined OLIF group at 1 week (30.9 ± 2.0 vs. 33.6 ± 2.1, *P* < 0.001) and 3 months (18.2 ± 2.4 vs. 20.7 ± 2.6, *P* < 0.001), but there was no significant difference in ODI at 2 years (14.6 ± 1.8 vs. 14.4 ± 2.1, *P* = 0.716). Patients in the standalone OLIF group suffered less leg pain at 3 months (1.5 ± 0.7 vs. 2.9 ± 0.9, *P* < 0.001) and 2 years (1.2 ± 0.7 vs. 2.2 ± 0.5, *P* < 0.001). The clinical outcomes are summarized in Table 2 and Figs. 2-3.

**Table 2:** Comparison of clinical outcomes between standalone and combined OLIF

	OLIF+fixation	Standalone OLIF	P
VAS (leg pain)			
Pre-op	5.6 ± 1.0	5.8 ± 0.7	0.275
Post-op 1 week	3.2 ± 0.8	3.0 ± 0.6	0.228
Post-op 3 months	2.9 ± 0.9	1.5 ± 0.7	< 0.001
Post-op 2 years	2.2 ± 0.5	1.2 ± 0.7	< 0.001
ODI			
Pre-op	44.8 ± 3.3	44.1 ± 2.6	0.342
Post-op 1 week	33.6 ± 2.1	30.9 ± 2.0	< 0.001
Post-op 3 months	20.7 ± 2.6	18.2 ± 2.4	< 0.001
Post-op 2 years	14.4 ± 2.1	14.6 ± 1.8	0.716
Satisfaction			0.692
Satisfied	38 (92.7%)	28 (87.5%)	
Unsatisfied	3 (7.3%)	4 (12.5%)	

*ODI* Oswestry disability index, *VAS* visual analogue scale

### Radiological outcomes

There were no significant differences in ADH between the two groups during follow-up (all *P* > 0.05). PDH and FW was smaller in the combined OLIF group compared with the standalone OLIF group before and after surgery (all *P* < 0.05). There were significant differences in FH before surgery and at 1 week and 3 months between the two groups (all *P* < 0.05), but the difference disappeared by 2 years (*P* = 0.111) (Table 3).

**Table 3:** Comparison of radiological outcomes between standalone and combined OLIF

	OLIF+fixation	Standalone OLIF	P
ADH			
Pre-op	4.9 ± 1.6	6.8 ± 2.5	< 0.001
Post-op 1 week	12.8 ± 1.2	12.7 ± 0.7	0.737
Post-op 3 months	12.8 ± 1.5	12.6 ± 0.5	0.991
Post-op 2 years	12.5 ± 1.2	12.0 ± 0.4	0.055
PDH			
Pre-op	4.1 ± 2.0	6.1 ± 2.3	0.010
Post-op 1 week	8.7 ± 1.7	10.2 ± 1.2	< 0.001
Post-op 3 months	8.6 ± 1.7	10.1 ± 1.0	< 0.001
Post-op 2 years	8.3 ± 1.6	9.9 ± 1.2	< 0.001
FH			
Pre-op	12.7 ± 3.7	14.5 ± 1.9	0.009
Post-op 1 week	16.6 ± 4.0	18.7 ± 3.2	0.014
Post-op 3 months	16.6 ± 4.0	18.4 ± 3.3	0.032
Post-op 2 years	16.4 ± 3.9	17.8 ± 3.3	0.111
FW			
Pre-op	7.4 ± 2.3	8.6 ± 1.7	0.017
Post-op 1 week	9.6 ± 1.5	10.8 ± 1.8	0.018
Post-op 3 months	9.4 ± 2.2	10.7 ± 1.8	0.011
Post-op 2 years	9.4 ± 2.2	10.6 ± 1.7	0.016
Fusion rate at 3 months			0.669
GradeI	35 (85.4%)	27 (84.4%)	
Grade II	4 (9.8%)	2 (6.3%)	
Grade III	2 (4.9%)	3 (9.4%)	
Grade IV	0	0	
Fusion rate at 2 years			0.066
Grade I	41 (100.0%)	30 (93.8%)	
Grade II	0	2 (6.2%)	
Grade III	0	0	
Grade IV	0	0	

*ADH* anterior disc height, *PDH* posterior disc height, *FH* foraminal height, *FW* foraminal width

Cage subsidence occurred in 7.3% (3/41) and 7.3% (3/41) of the patients at 3 and 24 months, respectively, in the combined OLIF group, compared with 6.3% (2/32) and 15.6% (5/32), respectively, in the standalone OLIF group at the same time points (*P* = 0.287). There was no cage retropulsion in both groups at 2 years.

The fusion rate was 85.4%(35/41) in the combined OLIF group and 84.4% (27/32) in the standalone OLIF group at 3 months(*P* = 0.669). At 24 months, the fusion rate was 100.0% in the combined OLIF group and 93.8% (30/32) in the standalone OLIF group (*P* = 0.066).

### Complications

**Fig. 4:**
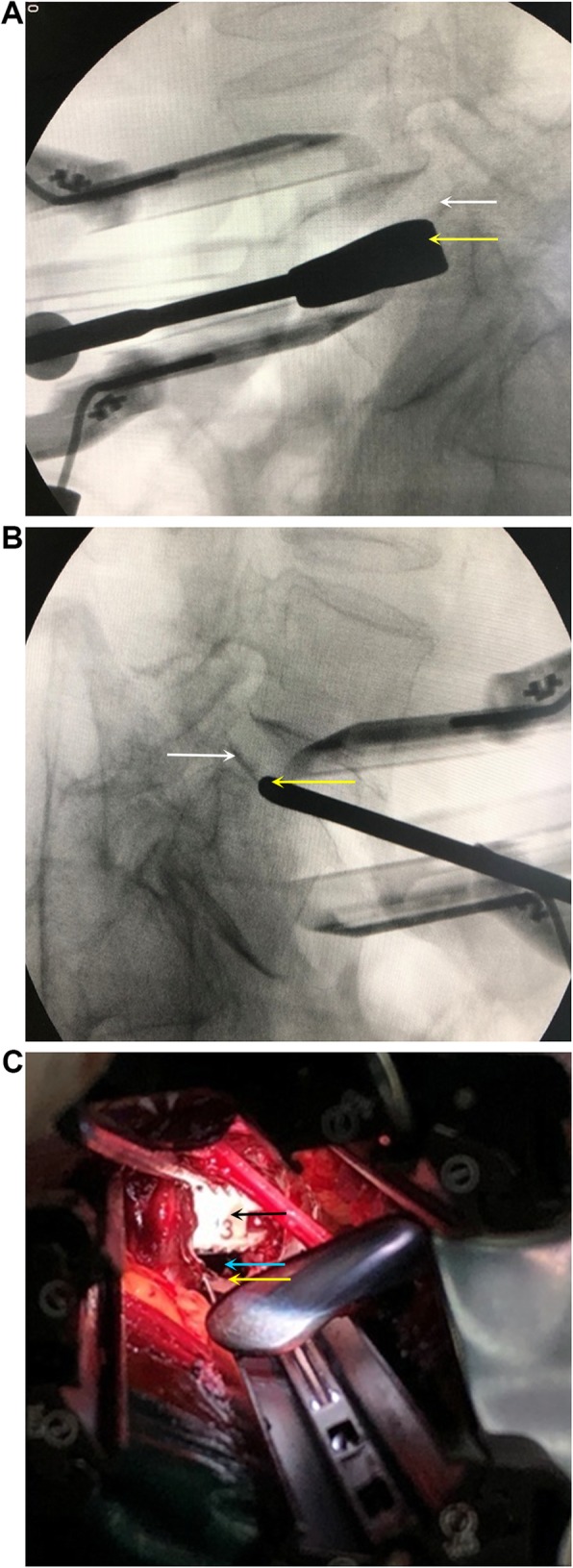
Intraoperative endplate damage. **a** White arrow: normal superior endplate of L5. Yellow arrow: the trial mold breaks in the vertebral body. **b** White arrow: normal superior endplate of L5. Yellow arrow: the trial mold breaks through the cortex of the endplate. **c** Yellow arrow: normal superior endplate of the vertebral body. Blue arrow: a cavity between the lower margin of the cage and the upper endplate of the vertebral body after the endplate is damaged and collapsed. The cavity was filled with DBM. White arrow: the cage

The total complication rate was 26.8% (11/41) in the combined OLIF group and 25.0% (8/32) in standalone OLIF group (Table 4). In the combined OLIF group, five patients had transient sympathetic injury and leg numbness, but they needed no specific treatment and recovered spontaneously within 3 months. Three cases of L4 segmental artery injury were noticed intraoperatively and immediate hemostasis was achieved using a hemoclip. Three cases of L4/5 endplate injury were noticed intraoperatively (Fig. 4). In the standalone OLIF group, four patients(L4/5) complained of leg pain or numbness at 1 day after OLIF. Two cases of L4 segmental artery injury were noticed intraoperatively and immediate hemostasis was achieved using a hemoclip. Two cases of L4/5 endplate injury were noticed intraoperatively. None of the patients in either group had intraoperative dural tear, screw malposition, transient erebrospinal fluid leak, infection, adjacent segment disease, deep vein thrombosis, or cage retropulsion.

**Table 4:** Complications after standalone and combined OLIF

	OLIF+fixation	Standalone OLIF	P
Intraoperative			
Segmental artery injury	3 (7.3%)	2 (6.3%)	0.857
Endplate damage	3 (7.3%)	2 (6.3%)	0.857
Post-operative			
Leg weakness	5 (12.2%)	4 (12.5%)	0.969
Sympathetic chain injury	5 (12.2%)	4 (12.5%)	0.969
Cage subsidence	3 (7.3%)	5 (15.6%)	0.287

## Discussion

OLIF has been found to result in a 30.2% median increase in the cross-sectional area of the dural sac [16] and a 30.0% average increase in the neural foramen area [17]. The results suggest that the standalone OLIF group achieved better clinical outcomes in the early postoperative term (1 week and 3 months), but the differences disappeared by 2 years. The probable reason is that standalone OLIF does not invade the muscle groups on both sides of the spine and does not cause intra muscular hematoma, leading to better early recovery [18]. The difference disappeared by 2 years once the muscles and hematoma healed in the combined OLIF group.

In the present study, the rate of cage subsidence in the standalone OLIF was 15.6% while it was 7.3% in the combined OLIF group. Zeng et al. [7] reported a rate of cage subsidence with standalone OLIF of 36.3%, higher than in the combined OLIF group (29.9%). Good clinical results of the OLIF without additional posterior pedicle screw fixation have been reported, but an indication was the absence of endplate damage [8–11]. Although the sample size was small in the present study, our results are supported by Zeng et al. [7] and the main reason for cage subsidence is probably endplate damage during surgery.

In total, there were three cases of cage subsidence in the standalone OLIF group and five in the combined OLIF group. All of them had a T score < − 2.5 on DEXA. Standalone OLIF inpatients with mild lumbar spondylolisthesis could be associated with a concern about complications, especially cage subsidence. Based on the present study, a T score warning line of <− 2.5 or body mass index (BMI) ≥30 kg/m^2^ could be associated with worse outcomes of standalone OLIF [19]. Of course, cage subsidence may occur after OLIF combined with pedicle screw fixation as well, but theoretically pedicle screw fixation could add more protection. Patients who are osteoporotic or obese might be better undergoing combined rather than standalone OLIF. On the other hand, for patients without those risk factors, standalone OLIF could be enough. Formal analyses of T values and BMI should be performed in future studies. In addition, the three patients with cage subsidence in the standalone OLIF group were elderly (88, 81, and 77 years of age). Of course, osteoporosis is associated with age, and we cannot rule out which factor is more important based on three cases. Nevertheless, other authors reported similar results. Li et al. [20] and Lee et al. [21] showed that lower BMD could result in cage migration. Pan et al. [22] and Lee et al. [23] reported that BMI of patients with cage retropulsion was higher than that in those without. Future studies could examine whether additional posterior pedicle screw fixation could be applied in the operation.

Lin et al. [24] evaluated 52 patients who underwent standalone OLIF without posterior instrumentation and reported a fusion rate of 81.9% at > 12 months after surgery when assessed by CT. Kim et al. [25] reported a 12-month fusion rate of 92.9% in 29 OLIF patients with posterior pedicle screw fixation when assessed with CT. In this study, we found that the low fusion rate in both OLIF group was related to cage subsidence, and the poor clinical effect was mostly inpatients who suffered from cage subsidence. The pedicle screw and the rod system are widely accepted and used to achieve stable and rigid fixation in patients undergoing fusion surgery, but there was no difference in fusion rate between the two groups, at least within 2 years of follow-up. The reason could be that despite the fact that standalone OLIF is more unstable compared with OLIF combined with screw implantation, the bilateral muscles remain intact with standalone OLIF, which could help stabilize the spine. Therefore, both methods achieved a similar fusion rate.

**Fig. 5:**
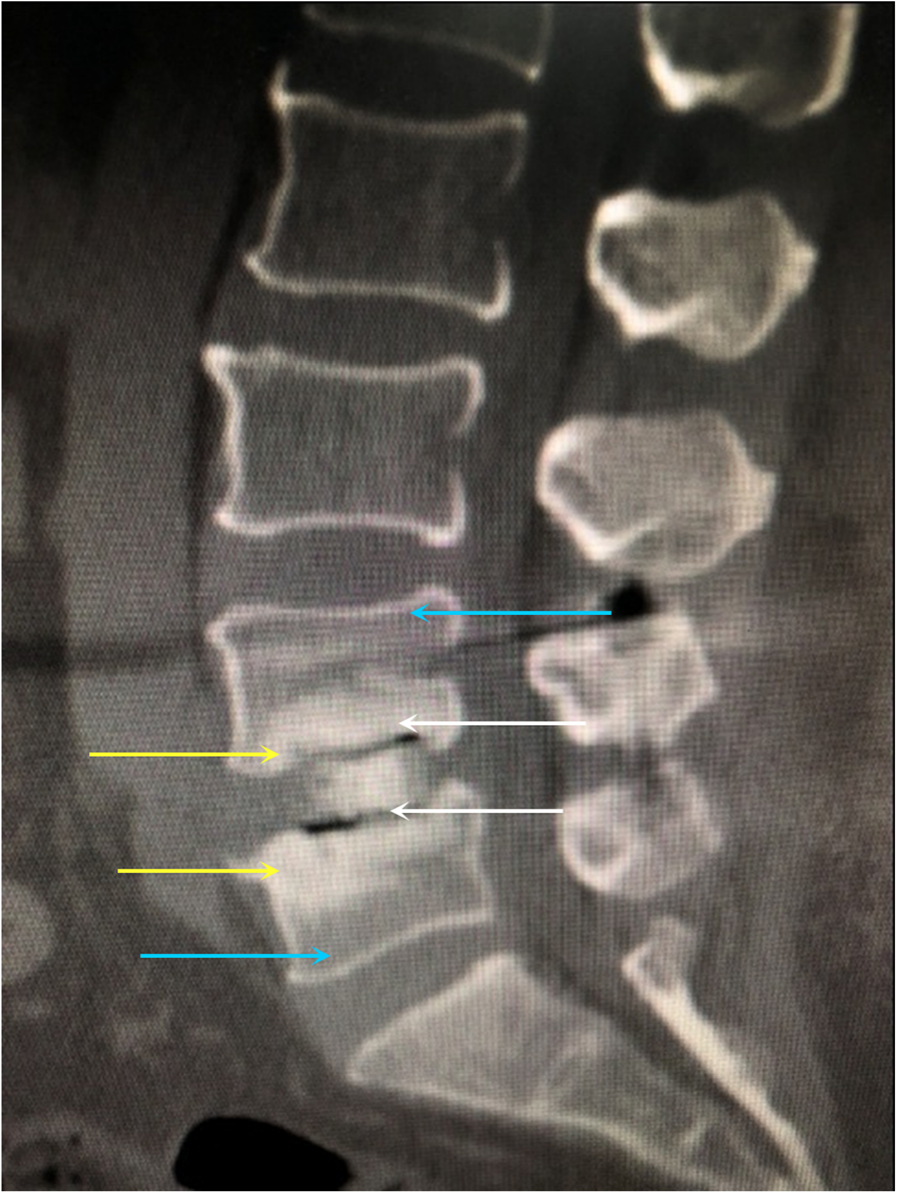
Postoperative spine CT scan. There was potential translucency present at top and bottom of graft(L4/5 cage). There were vertebral compression fractures in both lower endplate and vertebral body of the L4 and upper endplate and vertebral body of the L5. The portion between the two white arrows is the transparent strip. The blue arrow indicates the normal cancellous bone manifestation of the vertebral body. The orange arrow indicates the “hardening”that occurs after a compressive fracture of the cancellous bone inside the vertebral body

The results suggest that cage subsidence and adjacent vertebral fractures could be mutually causal. Endplate damage further causes trabecular bone fracture in the vertebral body, causing the vertebral body to be weakly supported, resulting in cage subsidence. Before calcification of vertebral hematoma, vertebral trabecular fracture and cage subsidence form a vicious circle. The intervertebral endplate and the cage are slightly moving, resulting in a decrease in the fusion rate (Fig. 5). Until the cancellous bone of the vertebral body is compressed to a certain density and reaches the support cage strength, a stable state is reached and the artificial bone in the cage ensures fusion when the growth of the upper and lower vertebral bodies starts. Micro-fractures in the vertebral body can produce hematoma, and the patient can feel pain and discomfort [18].

Despite the fact that fusion has been shown to achieve similar outcomes to those of conservative approaches [26], surgery was attempted in the patients included in the present study because those patients had failure to at least 6 months of conservative treatments. In addition, direct decompressions were not performed. Indeed, all patients included here suffered from grade I lumbar spondylolisthesis, and the instability of the lumbar spine mainly causes low back pain and only mild lower limb symptoms. For this kind of condition, OLIF can be used, and the clinical results are generally satisfactory. But for patients with concomitant conditions such as lumbar spinal stenosis or lumbar disc herniation, the lower limb symptoms may be more significant, therefore, and standalone OLIF cannot be used. The reason for using OLIF combined with pedicle screw fixation is that for osteoporosis patients, adding posterior internal fixation can reduce cage subsidence and increase the early fusion rate. Of course, the addition of posterior internal fixation will increase the economic burden on the patients. Therefore, for patients without osteoporosis or obesity, standalone OLIF is probably enough and should avoid damage to the back muscles and improves recovery, but additional studies are necessary to confirm those results. In addition, whether direct decompression could provide additional benefits should be examined.

Demineralized bone matrix (DBM) is a key factor in promoting fusion, and both groups received this growth factor, limiting the intergroup variability. In elderly patients with osteoporosis who received posterior pedicle screw fixation, the use of DBM increases the local stability and avoids compression fracture of the endplate caused by the cage, leading to a higher fusion rate. In this study, no allograft, autograft, or other bone substitute was used, and it should be examined in the future whether their use result in similar outcomes.

The present study has limitations. There trospective design introduces a degree of uncertainty due to missing and erroneous data. Especially, imaging examinations were performed routinely and the exact parameters were at the radiologists’ discretion at the time of examination, possibly introducing some bias in the measurements. Second, the small sample size likely affects the power of the statistical analysis of the demographics and radiological parameters. Third, the follow-up was short because OLIF has been introduced only a short time ago. Finally, even if the surgeons share a common education and training and regularly assist each other with cases and have a long history of cooperation, the patients were from two hospitals, possibly introducing bias. The possibilty of proof lies within a future prospective study, preferably an RCT.

## Conclusion

Standalone OLIF may achieve equivalent clinical and radiological outcomes compared to OLIF combined with fixation for spondylolisthesis. The rate of complication was similar between the two groups. Standalone OLIF does not invade the paraspinal muscle groups, possibly leading to better clinical effect and faster recovery, saving social and personal medical costs. Patients who are osteoporotic might be better undergoing combined rather than standalone OLIF.

## Data Availability

The datasets used and/or analysed during the current study available from the corresponding author on reasonable request.

## Abbreviations

OLIF: Oblique lateral interbody fusion
VAS: Visual analog scale
ODI: Oswestry Disability Index
DH: Disc heights
FH: Foraminal height
FW: Foraminal width
DSD: Degenerative lumbar spine disease
LLIF: Lateral lumbar interbody fusion
ALIF: Anterior lumbar interbody fusion
AA: Abdominal aorta
PM: Psoas muscle
BMD: Bone mineral density
CT: Computed tomography

## References

[1] Ravindra VM, Senglaub SS, Rattani A, Dewan MC, Hartl R, Bisson E (2018). Degenerative lumbar spine disease: estimating global incidence and worldwide volume. Global Spine J.

[2] Rhee JM, Schaufele M, Abdu WA (2006). Radiculopathy and the herniated lumbar disc. Controversies regarding pathophysiology and management. J Bone Joint Surg Am.

[3] Gregory DS, Seto CK, Wortley GC, Shugart CM (2008). Acute lumbar disk pain: navigating evaluation and treatment choices. Am Fam Physician.

[4] Silvestre C, Mac-Thiong JM, Hilmi R, Roussouly P (2012). Complications and morbidities of mini-open anterior retroperitoneal lumbar Interbody fusion: oblique lumbar Interbody fusion in 179 patients. Asian Spine J.

[5] Hynes R. Oblique lateral interbody fusion (OLIF) technique and complicationsin 457 levels L1 to S1. In: International society for the advancement of spinesurgery. Florida; 2014.

[6] Davis TT, Hynes RA, Fung DA, Spann SW, MacMillan M, Kwon B (2014). Retroperitoneal oblique corridor to the L2-S1 intervertebral discs in the lateral position: an anatomic study. J Neurosurg Spine.

[7] Zeng ZY, Xu ZW, He DW, Zhao X, Ma WH, Ni WF (2018). Complications and prevention strategies of oblique lateral Interbody fusion technique. Orthop Surg.

[8] Zhang JF (2017). Clinical value of one-level oblique lateral interbody fusion in the treatment of degenerative lumbar disc diseases. ZhonghuaGuKeZaZhi.

[9] Pimenta L, Marchi L, Oliveira L, Coutinho E, Amaral R (2013). A prospective, randomized, controlled trial comparing radiographic and clinical outcomes between stand-alone lateral interbody lumbar fusion with either silicate calcium phosphate or rh-BMP2. J Neurol Surg A Cent Eur Neurosurg.

[10] Kwon YK, Jang JH, Lee CD, Lee SH (2014). Fracture of the L-4 vertebral body after use of a stand-alone interbody fusion device in degenerative spondylolisthesis for anterior L3-4 fixation. J Neurosurg Spine.

[11] Mehren C, Mayer HM, Zandanell C, Siepe CJ, Korge A (2016). The oblique anterolateral approach to the lumbar spine provides access to the lumbar spine with few early complications. Clin Orthop Relat Res.

[12] Ohtori S, Orita S, Yamauchi K, Eguchi Y, Ochiai N, Kishida S (2015). Mini-open anterior retroperitoneal lumbar Interbody fusion: oblique lateral Interbody fusion for lumbar spinal degeneration disease. Yonsei Med J.

[13] Donnally IC, Varacallo M. Lumbar Degenerative Disk Disease. In: StatPearls: Treasure Island (FL); 2018. 28846354

[14] Lim TH, Kwon H, Jeon CH, Kim JG, Sokolowski M, Natarajan R (2001). Effect of endplate conditions and bone mineral density on the compressive strength of the graft-endplate interface in anterior cervical spine fusion. Spine (Phila Pa 1976).

[15] Bridwell KH, Lenke LG, McEnery KW, Baldus C, Blanke K (1995). Anterior fresh frozen structural allografts in the thoracic and lumbar spine. Do they work if combined with posterior fusion and instrumentation in adult patients with kyphosis or anterior column defects?. Spine (Phila Pa 1976).

[16] Fujibayashi S, Hynes RA, Otsuki B, Kimura H, Takemoto M, Matsuda S (2015). Effect of indirect neural decompression through oblique lateral interbody fusion for degenerative lumbar disease. Spine (Phila Pa 1976).

[17] Sato J, Ohtori S, Orita S, Yamauchi K, Eguchi Y, Ochiai N (2017). Radiographic evaluation of indirect decompression of mini-open anterior retroperitoneal lumbar interbody fusion: oblique lateral interbody fusion for degenerated lumbar spondylolisthesis. Eur Spine J.

[18] Yang Y, Zhang L, Liu B, Pang M, Xie P, Chen Z (2017). Hidden and overall haemorrhage following minimally invasive and open transforaminal lumbar interbody fusion. J Orthop Traumatol.

[19] Wang YP, An JL, Sun YP, Ding WY, Shen Y, Zhang W (2017). Comparison of outcomes between minimally invasive transforaminal lumbar interbody fusion and traditional posterior lumbar intervertebral fusion in obese patients with lumbar disk prolapse. Ther Clin Risk Manag.

[20] Li H, Wang H, Zhu Y, Ding W, Wang Q (2017). Incidence and risk factors of posterior cage migration following decompression and instrumented fusion for degenerative lumbar disorders. Medicine (Baltimore).

[21] Lee JG, Lee SM, Kim SW, Shin H (2013). Repeated migration of a fusion cage after posterior lumbar interbody fusion. Korean J Spine.

[22] Pan FM, Wang SJ, Yong ZY, Liu XM, Huang YF, Wu DS (2016). Risk factors for cage retropulsion after lumbar interbody fusion surgery: series of cases and literature review. Int J Surg.

[23] Lee DY, Park YJ, Song SY, Jeong ST, Kim DH (2018). Risk factors for posterior cage migration after lumbar Interbody fusion surgery. Asian Spine J.

[24] Lin JF, Iundusi R, Tarantino U (2010). Intravertebral plate and cage system via lateral trajectory for lumbar interbody fusion-a novel combined device. Spine J.

[25] Kim JS, Choi WS, Sung JH (2016). 314. Minimally invasive oblique lateral interbody fusion for L4-5: clinical outcomes and perioperative complications. Neurosurgery.

[26] Mannion AF, Brox JI, Fairbank JC (2013). Comparison of spinal fusion and nonoperative treatment in patients with chronic low back pain: long-term follow-up of three randomized controlled trials. Spine J.

